# Generation and agglomeration behaviour of size-selected sub-nm iron clusters as catalysts for the growth of carbon nanotubes

**DOI:** 10.3762/bjnano.2.80

**Published:** 2011-11-01

**Authors:** Ravi Joshi, Benjamin Waldschmidt, Jörg Engstler, Rolf Schäfer, Jörg J Schneider

**Affiliations:** 1 Department of Chemistry, Eduard-Zintl-Institute, Inorganic Chemistry, Technische Universität Darmstadt, Petersenstr. 18, 64287 Darmstadt, Germany, Fax: +49-6151-16-3470, Tel: +49-6151-16-3225; 2 Department of Chemistry, Eduard-Zintl-Institute, Physical Chemistry, Technische Universität Darmstadt, Petersenstr. 20, 64287 Darmstadt, Germany, Fax: +49-6151-16-6024, Tel: +49-6151-16-2498

**Keywords:** carbon nanotubes, CNT growth, metal clusters, size selected clusters

## Abstract

Mass-selected, ligand-free Fe*_N_* clusters with *N* = 10–30 atoms (cluster diameter: 0.6–0.9 nm) were implanted into [Al@SiO*_x_*] surfaces at a low surface coverage corresponding to a few thousandths up to a few hundredths of a monolayer in order to avoid initial cluster agglomeration. These studies are aimed towards gaining an insight into the lower limit of the size regime of carbon nanotube (CNT) growth by employing size-selected sub-nm iron clusters as catalyst or precatalyst precursors for CNT growth. Agglomeration of sub-nm iron clusters to iron nanoparticles with a median size range between three and six nanometres and the CNT formation hence can be observed at CVD growth temperatures of 750 °C. Below 600 °C, no CNT growth is observed.

## Introduction

Controlling the individual diameters of carbon nanotubes (CNTs) is still one of the major challenges in current CNT research, and it is particularly important as it determines crucially their physical and electronic properties. High quality single-walled and double-walled CNTs are currently prepared on a large scale with the aid of nm-sized transition metal catalysts, by using high-temperature chemical vapour deposition (CVD) techniques above 750 °C, despite the fact that several modifications of the CVD technique exist that allow growth processes under moderate conditions even below 400 °C [1–3]. Therefore obtaining more-selective catalysts that may allow reduction of the synthesis temperature in thermal CVD processes even further, while at the same time maintaining a high quality of the CNT material, would be a challenging goal. The introduction of defined metal-cluster catalysts with size-selected dimensions, deposited with a low surface coverage onto a suitable growth substrate to maintain their integrity, thus provides a new route into size-selective CNT growth. Moreover, this allows us to tackle the lower size limit of catalyst particles necessary for CNT growth. The smallest CNT diameter realized so far is 0.4 nm, which was found to be stable only in a template environment, either of a double walled CNT [4–5], or the interior of a porous zeolite AlPO_4_-5 single crystal [6]. Size-defined, sub-nm, ligand-free metal clusters would be an interesting synthetic alternative to obtain CNTs with controlled diameter. However, such small metal clusters can only be generated by gas-phase techniques, typically in a ligand-free environment as naked clusters. A controlled decrease in catalyst particle size below 1 nm and their deposition with a very low substrate surface coverage would allow studying of the lower size regime of such clusters and their catalytic activity in CNT growth. This could thus give information on the critical size of the catalyst particles and allow insight as to what extent catalyst agglomeration under thermal annealing of these particles occurs under typical CVD growth conditions. Larger catalyst particles in the size range of 3–10 nm can maintain their size throughout the growth process of CNTs, which typically operates at 750 °C or higher [7].

## Results and Discussion

**Figure 1:**
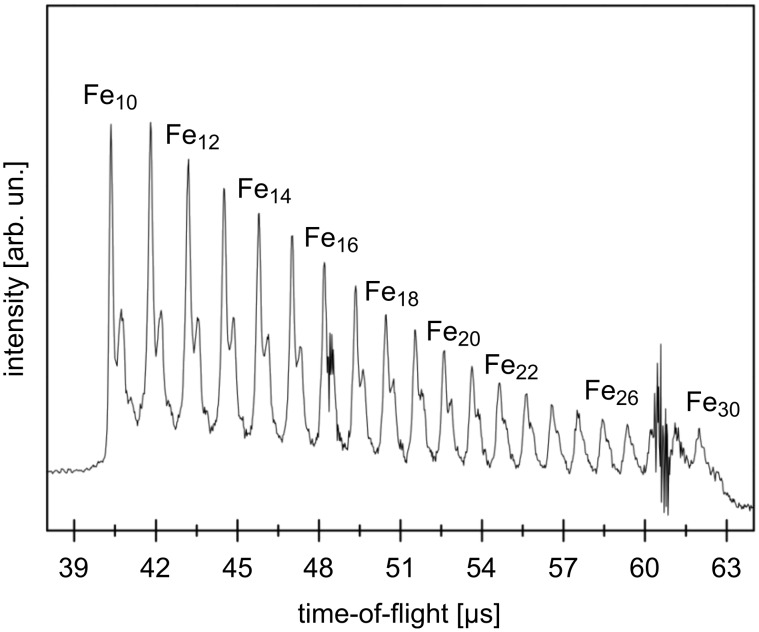
Time-of-flight mass spectrum of the selected cluster size distribution showing a pure iron cluster with 10 to 30 atoms but also oxide clusters, which appear in the right shoulder of the iron-cluster peaks. Clusters with 10 to 30 atoms were selected by applying a voltage pulse with an appropriate duration to the electrostatic mirror. The noise signal seen between Fe_28_ and Fe_29_ has an electronic origin and is due to the gating of the electrostatic mirror. The intensity of the iron species decreases with increasing cluster size. Beside pure iron clusters a minor quantity of metal oxide clusters is present in the molecular beam.

Herein we report our studies on the deposition of size-selected ligand-free iron clusters, in the sub-nm regime (Fe_10–30_: 0.6–0.9 nm size, Figure 1), onto a [Al@SiO*_x_*] substrate and their subsequent catalytic activity in the CVD growth of CNTs. The main goal of our studies is to unravel the behaviour of active, as deposited, ultrasmall naked metal catalyst particles under realistic growth conditions for CNTs. To the best of our knowledge these are the first experimental studies on CNT growth employing size-selected, sub-nm iron clusters as catalyst or precatalyst precursors. (A study of 3-D metal clusters as catalysts in CNT growth was reported in [8], however, no CNT growth was studied therein; CNT growth derived from nm-sized, ligand-stabilized mixed metal clusters as precatalysts was reported in [9].) Non-agglomerated naked iron clusters of 0.6–0.9 nm size were generated, mass-selected and surface-implanted at room temperature under ultrahigh vacuum [10] at a surface coverage in the range of a few thousandths up to a few hundredths of a monolayer on standard substrate grids for transmission electron microscopy (TEM). The kinetic energy of the as deposited iron clusters is about 2550 ± 150 eV, which is sufficient for their surface implantation [11].

Due to the substrate transfer to the CVD chamber the deposited iron clusters most likely become easily oxidized. However, the CNT growth takes place at high temperatures and in the presence of H_2_, which certainly reduces all of the formed oxide species that are accumulated. A 10 nm Al layer was deposited prior to cluster deposition on the SiO*_x_* grid. This thin Al barrier layer later ensures CNT growth [12]. Sintering of the iron catalyst during heating is minimized due to the low Tammann temperature of aluminium (194 °C), which prevents the iron particles from agglomeration and hence providing a good aluminium–iron interaction. Moreover, the deposited Al layer is also partially oxidized on top of the SiO*_x_* grid surface [12] and forms a stable alumina–catalyst interface, which stabilizes the deposited iron clusters further [13]. The [Fe*_N_*/Al@SiO*_x_*] grid substrates are then transferred under air to the CVD chamber and a water-assisted catalyzed CNT growth with ethylene as carbon source [12–13] is performed under different thermal annealing conditions (for the detailed experimental setup of the molecular beam and a description of the CVD apparatus see the Experimental section).

**Figure 2:**
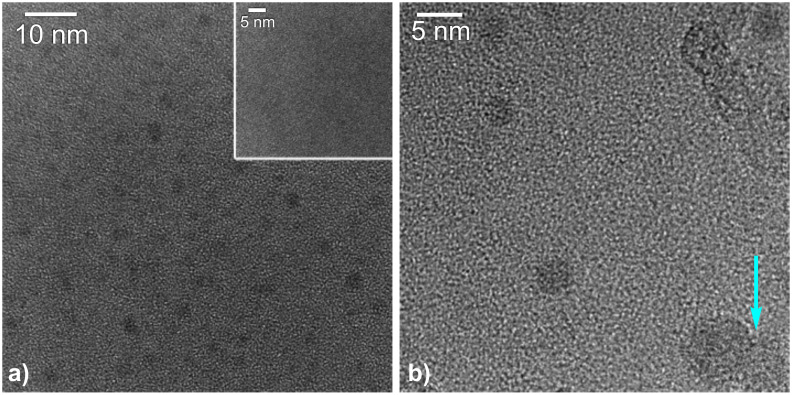
a) High resolution (HR)TEM micrographs of the products obtained after deposition of 0.6–0.9 sub-nm iron clusters and subsequent thermal annealing for 10 min at 750 °C. Inset in a): TEM micrograph of the grid surface after initial iron cluster deposition and thermal annealing up to a maximum temperature of 600 °C for 10 min; b) TEM of an isolated and a twinned crystalline catalyst particle (see lower right corner) obtained after cluster sintering of the 0.6–0.9 sub-nm iron clusters at 750 °C.

Cluster deposition followed by thermal annealing of size-selected clusters with a diameter of 0.6–0.9 nm and at a coverage of 3% of a monolayer on a [Al@SiO*_x_*] TEM grid up to temperatures of 600 °C did not lead to any detectable cluster agglomeration under high-resolution TEM conditions (note that iron clusters in a size regime of 0.6–0.9 nm and 3% of a monolayer thickness are close to or below the detection limit of the electron microscope). However, annealing these substrate samples up to 750 °C for 10 min, which are typical CVD growth parameters, led to an agglomeration of the small sub-nm iron clusters to form iron nanoparticles and hence allowing their subsequent detection under the microscope (Figure 2). This cluster growth process occurs by Ostwald ripening, which takes place as a fast process in a matter of minutes at this temperature [14]. Our findings are in accordance with the stability of size-selected Au clusters pinned on graphite for which strong cluster agglomeration above 600 °C was shown [11], indicating that a combination of diffusion controlled agglomeration and Ostwald ripening of Fe clusters becomes significant at temperatures above that particular temperature [14].

**Figure 3:**
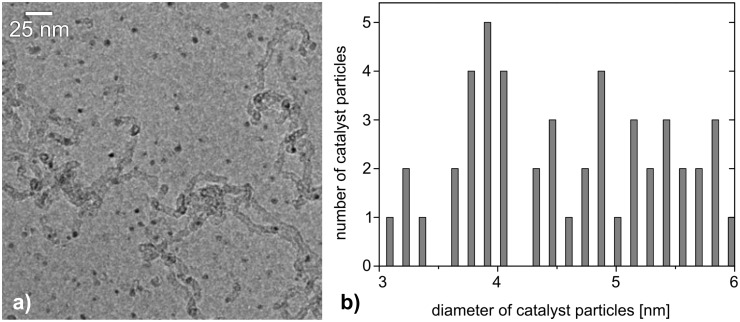
a) TEM of iron catalyst particles and CNTs formed from size-selected 0.6–0.9 nm iron clusters after CNT growth at 750 °C. The catalyst particles are homogeneously distributed, and scattered CNT growth is also observed. b) Histogram of the size distribution of the iron catalyst particles formed from the size-selected 0.6–0.9 nm iron clusters at 750 °C. 86% of all of the counted particles are shown. The remaining 14% are well below or above the shown size distribution (1 nm and 9–20 nm).

**Figure 4:**
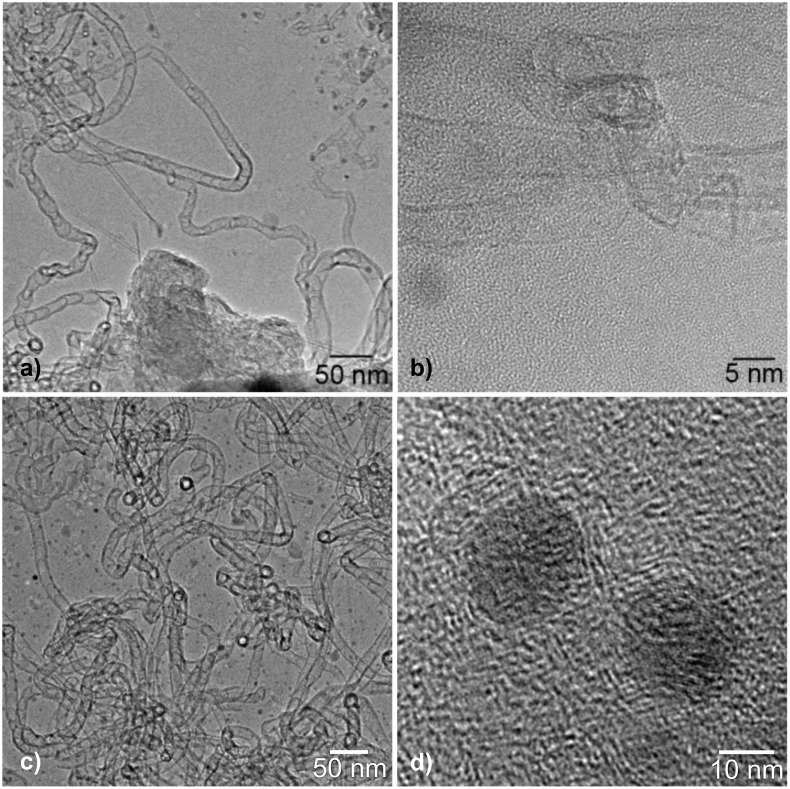
TEM pictures of CNT formation at isolated iron nanoparticles originated from 0.6–0.9 nm sub-nm iron particles; a) TEM overview of CNTs formed from isolated iron nanoparticles; b) HRTEM of double walled CNTs at 750 °C formed from the same catalyst particles; c) as a), showing mainly bamboo-shaped multiwalled CNTs of ca. 20 nm diameter from the same sample; d) HRTEM micrograph of crystalline iron nanoparticles obtained from 0.6–0.9 nm size-selected iron clusters by extensive agglomeration processes.

The size of the agglomerated clusters can be estimated on a thermodynamic basis [15], however, this does not include the influence of the cluster–substrate surface interaction on the surface energy of iron, which may also affect the cluster size. Nevertheless with the volume of the unit cell of 23.5 Å^3^ and the surface energy of 2.4 J/m^2^ for α-iron [16], a mean diameter of 3.0 nm with a standard deviation of 1.7 nm was obtained for a substrate temperature of 750 °C, which is in good agreement with the nanoparticle diameter as observed experimentally by TEM (Figure 3 and see discussion below, and Figure 4).

Subsequently employing CNT growth conditions (C_2_H_2_/water/H_2_ [12–13,17]) with these sub-nm iron clusters as catalysts at 600 °C did not lead to any CNT formation on the [Al@SiO*_x_*] substrates, meaning that at 600 °C the deposited iron clusters are obviously too small or inactive for CNT growth. The observation that CNT growth is possible at temperatures as low as 350 °C [18], although with significantly larger iron catalyst particles of 3–5 nm in diameter, is not contradictory to our process conditions. Cluster implantation in combination with substrate interaction of the sub-nm iron clusters the [Al@SiO*_x_*] substrate surface is effective in preventing cluster sintering. This fact is very helpful since small sub-nm clusters are already significantly mobile due to the reduced Tammann temperature of the iron clusters (Tammann temperature of 631 °C for bulk iron and 269 °C for iron atoms at defects [19]). For the as deposited sub-nm iron clusters studied herein, the mobility should be even higher at elevated temperatures, necessitating a high-energy surface implantation for these mobile fragments [20–21].

Raising the temperature of the [Fe*_N_*/Al@SiO*_x_*] samples from 600 to 750 °C nevertheless led to significant cluster agglomeration of the as deposited 0.6–0.9 nm iron clusters to form larger crystalline aggregates (Figure 2b). The high mobility of the iron clusters under thermal growth conditions at 750 °C is obvious from the observation of twinned 5 nm catalyst particles as well as even larger aggregates, which are occasionally found during the TEM observations (Figure 2b). Depending on the activation barrier of the diffusion process, a temperature increase of 150 °C can lead to a significant increase in the diffusion length of the clusters [22]. Employing water-assisted CVD growth conditions (C_2_H_2_/water/H_2_ [12–13,17], *T* = 750 °C) on [Fe*_N_*/Al@SiO*_x_*] substrates indeed led to a formation of CNTs catalyzed by the aggregated iron particles. These aggregated catalyst particles show a size distribution with a total of 86% of the counted catalyst particles being between 3–6 nm in diameter (Figure 3b). This growth accounts for a three- to five-fold increase of the initial cluster size up to a nominal Fe*_N_* cluster size between 1250–3750 Fe atoms, assuming a spherical 3-D cluster shape. The remaining iron clusters are significantly larger in size due to further agglomeration processes. During CNT growth, adsorbed hydrocarbons, hydrogen gas and water are present in a significant excess on the iron cluster surface, and thus these “surfactants” will certainly modify the surface energy of the catalyst clusters [23], and may have an additional profound influence, aside from the previously discussed reasons for cluster mobility and stability on the substrate surface, offering a reasonable explanation for this enhanced cluster growth. For instance, these effects may lead to further cluster sintering and explain the observation of larger cluster particles and the subsequent growth of larger diameter CNTs from these. Consequently the diameter of the CNTs in the water-assisted CVD growth varies between 5 nm for isolated, double-walled CNTs and reaches a maximum of up to about 12–15 nm for larger entanglements of multiwalled CNTs. The larger CNTs exhibit a distinct bamboo-like structure showing traversal of the inner cavity by graphitic layers that cap the inner tubes (Figure 4c). Different CNT morphologies exist in parallel to one another in similar samples, indicating the presence of differently sized catalytically active iron nanoparticles in the CNT growth process. Finally, it should be noted that we have also performed cluster-deposition experiments with larger (>1 nm) iron clusters, employing a cluster deposition density of up to a complete monolayer in terms of the substrate surface coverage. Similar agglomerated iron particles and comparable CNT formation was observed as it was in experiments with the smaller iron clusters at low initial cluster surface coverage.

## Conclusion

Nanometer-sized catalytically active iron nanoparticles are formed under typical CVD process conditions from mass-selected sub-nm iron clusters. The iron clusters were deposited on SiO*_x_*-coated copper TEM grids and then thermally annealed to achieve the CVD process conditions necessary for CNT growth. Due to the temperatures necessary for CNT growth, significant cluster agglomeration was observed, leading to a growth of the as deposited sub-nm clusters to form larger particles with a mean diameter of 3.0 ± 1.7 nm. This agglomeration occurs even though the sub-nm clusters were implanted into the SiO*_x_* surface to restrict their lateral mobility on the substrate surface. From our experiments it became evident that significant cluster growth on oxide surfaces due to the high synthesis temperatures necessary for CNT growth seems unavoidable when such sub-nm small clusters are used. In order to avoid this, a marked reduction in CNT growth temperature, to below 600 °C, is necessary.

## Experimental

### Size-selected cluster synthesis

**Figure 5:**
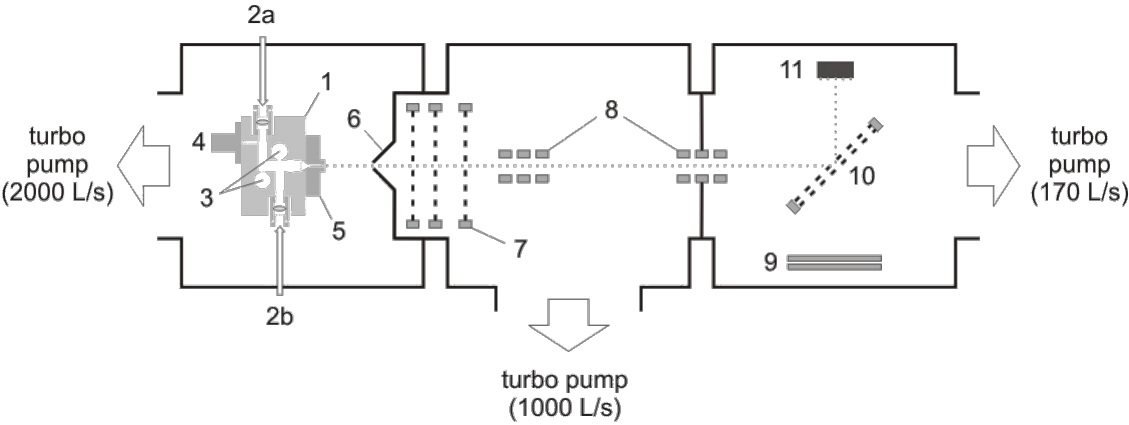
Setup of the iron cluster deposition system used in the deposition experiments.

A schematic overview of the vacuum system with a base pressure in the range of 10^−8^ mbar is shown in Figure 5. Iron clusters were produced by a bimetallic cluster source (1) based on the laser vaporization technique: The fundamental-wavelength beam of a Nd:YAG laser (2a) with a pulse width of 8 ns and an intensity of typically 100 mJ/pulse was focused on an Fe rod (3), synchronized with a pulsed He flow (4) at a stagnation pressure of 6 bar and a duration of approximately 300 µs. Since only pure iron clusters were investigated in the present work the second Nd:YAG laser (2b) was switched off. The plasma generated by the laser pulse was rapidly cooled down by collisions with He atoms, thereby forming clusters. The cluster–helium mixture was then injected through a nozzle (5) into an ultrahigh vacuum apparatus. Passing a skimmer (6), positive ions in the molecular beam were accelerated by a Wiley–McLaren time-of-flight (TOF) unit (7), guided by two Einzel lenses (8), and detected with a tandem microchannel plate (9). Mass spectra were obtained in a linear TOF mode with a rotatable 90° electrostatic mirror (10) aligned to the microchannel plate detector. For the deposition experiments, mass selection was performed by application of a high voltage pulse to the 90° mirror, which was aligned to the target (11). The incident energy of the mass-selected clusters was 2550 eV with an energy spread of approximately 150 eV (FWHM).

### Chemical vapour deposition of CNTs

A 10 nm thick aluminium buffer layer was deposited by means of either thermal evaporation (heating Al in a boron nitride crucible with a tungsten filament) or electron beam evaporation onto a commercial TEM grid (SiO*_x_*; Fa. Plano, Wetzlar). Aluminium buffer layer (10 nm) deposition was monitored by means of a quartz crystal microbalance (Cressington MTM 10). After deposition the TEM grids were transferred into the iron-cluster-source apparatus and the size-selected iron clusters were deposited. After cluster deposition, the TEM grids were placed in the CVD reactor and CNTs were synthesized by a water-assisted chemical vapor deposition method [6–7]. At the growth temperature of 750 °C, the ethylene (100 sccm) precursor gas flow was started and a small amount of the carrier gas was bubbled through a water bubbler in order to carry a defined amount of water vapor along with it. The amount of water vapor was monitored by using a commercial water vapour sensor built in the line (Mitchel instruments). The gas flow was controlled by using commercially available mass flow controllers (MKS instruments, Munich, Germany).

TEM characterization of the metal catalysts and the CNTs was performed with a Technai F20 (HRTEM) instrument with a field-emission gun, operated at an acceleration voltage of 20 kV.

## References

[1] Maruyma S, Koijima R, Miyauchi Y, Chaiashi S, Kohnao M (2002). Chem Phys Lett.

[2] Liao H W, Hafner J H (2004). J Phys Chem B.

[3] Min Y-S, Bae E-J, Oh B S, Kang D, Park W J (2005). J Am Chem Soc.

[4] Qin L-C, Zhao X, Hirahara K, Miyamoto Y, Ando Y, Iijima S (2000). Nature.

[5] Guan L, Suenaga K, Iijima S (2008). Nano Lett.

[6] Wang N, Tang Z K, Li G D, Chen J S (2000). Nature.

[7] Schäffel F, Kramberger C, Rümmeli M H, Kaltofen R, Grimm D, Grüneis A, Mohn E, Gemming T, Pichler T, Büchner B (2007). Phys Status Solidi A.

[8] Inoue S, Maruyama S (2008). Jpn J Appl Phys.

[9] An L, Owens J M, McNeil L E, Liu J (2002). J Am Chem Soc.

[10] Turra M, Waldschmidt B, Kaiser B, Schäfer R (2008). Rev Sci Instrum.

[11] Yin F, Xirouchaki C, Guo Q, Palmer R E (2005). Adv Mater.

[12] Joshi R, Engstler J, Houben L, Bar Sadan M, Weidenkaff A, Mandaliev P, Issanin A, Schneider J J (2010). ChemCatChem.

[13] Joshi R, Schneider J J, Yilmazoglu O, Pavlidis D (2010). J Mater Chem.

[14] Kim S M, Pint C L, Amama P, Hauge R H, Maruyama B, Stach E A (2010). J Mater Res.

[15] Bulyarskii S V, Pyatilova O V, Tsygantsov A V, Basaev A S, Galperin V A, Pavlov A A, Shaman Y P (2010). Semiconductors.

[16] Yu J, Lin X, Wang J, Chen J, Huang W (2009). Appl Surf Sci.

[17] Hata K, Futaba D N, Mizuno K, Namai T, Yumura M, Iijima S (2004). Science.

[18] Cantoro M, Hofmann S, Pisana S, Scardaci V, Parvez A, Ducatai C, Ferrari A C, Blackburn A, Wang K Y, Robertson J (2006). Nano Lett.

[19] Moulijn J A, van Diepen A E, Kapteijn F (2001). Appl Catal, A.

[20] Iijima S, Ichihashi T (1986). Phys Rev Lett.

[21] Smith D J (1985). J Vac Sci Technol, B: Microelectron Process Phenom.

[22] Sushumna I, Ruckenstein E (1985). J Catal.

[23] Egelhoff W F, Steigerwald D A (1989). J Vac Sci Technol, A.

